# Low-Fluorescence Starter for Optical 3D Lithography of Sub-40 nm Structures

**DOI:** 10.1021/acsaom.3c00031

**Published:** 2023-05-12

**Authors:** Georgii Gvindzhiliia, Dmitry Sivun, Christoph Naderer, Jaroslaw Jacak, Thomas A. Klar

**Affiliations:** † Institute of Applied Physics, Johannes Kepler University Linz, 4040 Linz, Austria; ‡ Department of Medical Engineering, University of Applied Sciences Upper Austria, 4020 Linz, Austria

**Keywords:** STED-inspired lithography, direct laser writing, two-photon lithography, multicolor photoinhibition lithography, peripheral photoinhibition lithography

## Abstract

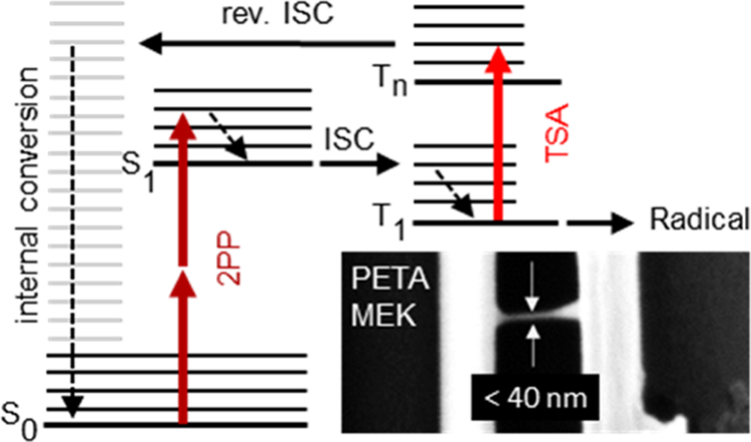

Stimulated emission depletion (STED) has been used to break the diffraction limit in fluorescence microscopy. Inspired by this success, similar methods were used to reduce the structure size in three-dimensional, subdiffractional optical lithography. So far, only a very limited number of radical polymerization starters proved to be suitable for STED-inspired lithography. In this contribution, we introduce the starter Michler’s ethyl ketone (MEK), which has not been used so far for STED-inspired lithography. In contrast to the commonly used 7-diethylamino-3-thenoylcoumarin (DETC), nanostructures written with MEK show low autofluorescence in the visible range. Therefore, MEK is promising for being used as a starter for protein or cell scaffolds in physiological research because the autofluorescence of DETC so far excluded the use of the green emission channel in multicolor fluorescence or confocal microscopy. In turn, because of the weak transitions of MEK in the visible spectrum, STED, in its original sense, cannot be applied to deplete MEK in the outer rim of the point spread function. However, a 660 nm laser can be used for depletion because this wavelength is well within the absorption spectrum of transient states, possibly of triplet states. We show that polymerization can be fully stopped by applying transient state absorption at 660 nm and that structure sizes down to approx. 40 nm in the lateral and axial directions can be achieved, which means 1/20 of the optical wavelength used for writing.

## 1 Introduction

Optical lithography is ubiquitous in micro- and nanoscale patterning. Two-photon optical lithography sticks out because of its intrinsic capability to write three-dimensional (3D) structures in a single run without the need for layer-by-layer stacking because of the vertical sectioning capability of two-photon absorption in microscopy^1^ and lithography.^2^ Using red or near-infrared laser pulses for two-photon excitation of polymerization starters also bears the advantage of using comparatively low photon energies compared to (extreme) UV or e-beam lithography. While the latter two are well suited for patterning crystalline semiconducting materials, they are often unsuitable for structuring soft materials such as organic semiconductors or biomaterials, considering their destructively high quantum energies. However, lithography based on multiphoton polymerization (MPP) is limited in reaching nanoscale structure sizes. Although subdiffractional lateral structuring has been realized due to the chemical nonlinearity imposed by the polymerization threshold, 50 nm structures are practically out of reach when using an 800 nm femtosecond laser for MPP.^3,4^

**Figure 1:**
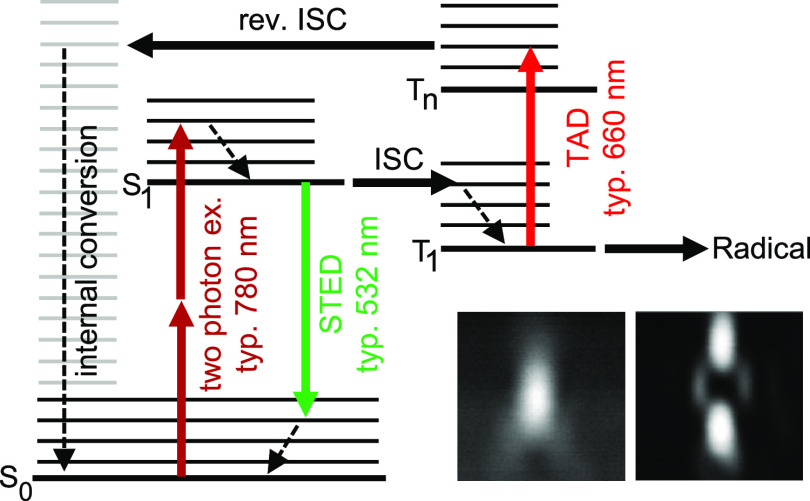
Jabłonski diagram of a photostarter. After two-photon excitation, the starter may proceed to some transient state T_1_ [for instance, to a triplet state via intersystem crossing (ISC)] from where it starts radical polymerization. This sequence can be intervened either within the singlet system by stimulated emission depletion (STED) in order to prevent the transfer to T_1_ or by transient absorption depletion (TAD) that pushes the starter into a higher state T*_n_* from where it returns to the singlet ground state, for instance via reverse ISC, followed by rapid internal conversion down to the ground state S_0_. The insets show the excitation PSF (780 nm, left) and the TAD PSF (660 nm, right) measured via backscattering from a 60 nm gold bead.

Motivated by subdiffractional optical microscopy using stimulated emission depletion (STED),^5,6^ optical two-photon lithography was boosted by similar techniques. The basic idea is to inhibit polymerization in the outer rim of a diffraction-limited point spread function (PSF) so that MPP only proceeds in the very center of the PSF. This yields an effective PSF, which can be substantially smaller than a diffraction-limited PSF. Several methods have been used to prevent polymerization in the exterior of the MPP excitation PSF. Similar to STED microscopy, where stimulated emission depletes the excited state of a fluorophore within typically 10-100 ps so that fluorescence (typically on an ns timescale) cannot occur anymore, the starters of a polymerization reaction can be depleted via STED before they proceed toward forming radicals, usually via the passage through one or several transient states such as a triplet state (Figure 1). This method, which we call STED lithography, was first realized by the Wegener group.^7,8^ Alternatively, one can wait until the starter has actually transferred to a transient state T_1_^9−15^ but prevent further reaction at this stage by transient absorption. An example would be a triplet–triplet absorption to a higher lying state T*_n_* from where it undergoes reverse intersystem crossing, followed by internal conversion to the ground state S_0_ (Figure 1).^16^ We stress that T_1_ and T*_n_* do not necessarily need to be triplet states, although, in radical polymerization, they often are triplets. In principle, any other intermediate transient state along the reaction path toward the initiation of radical polymerization that shows transient absorption may be suitable, too, if this transient absorption stops the reaction path. Examples are an intramolecular charge transfer state, a twisted biradical state within the singlet system,^17^ or an isomerization state.^18^ Hence, the abbreviation T_1_ should not necessarily refer to a triplet state but, more generally, to an energetic level of a transient species which can be excited to a state T*_n_* from where the molecule returns to the original ground state, hence preventing the start of polymerization or interrupting the growth of the polymer chain. We subsume all of these possibilities under the term “transient absorption depletion” (TAD) lithography. In both STED and TAD lithography, the depleting PSF needs to be arranged around the focal spot but must leave zero intensity in the center. This can be achieved by a so-called bottle beam, which is shown in the right inset of Figure 1 (the left inset shows the MPP excitation focus). Such a bottle-beam PSF is readily created by an annular phase delay of π inserted into the depleting beam before entering the objective lens.^19^ Typical structure sizes achieved so far are in the range of 50 nm both in the lateral^10,20,21^ and axial direction.^4^ Lateral feature sizes below 40 nm were reported recently,^15,22,23^ but no improvements in axial feature sizes were attempted in these studies.

Up to now, STED-inspired peripheral photoinhibition has been restricted to radical polymerization, mostly using acrylates or methacrylates as monomers. The number of radical starters for STED-inspired lithography is limited, as well. First, malachite green (MG) was used in TAD lithography.^9^ Then, isopropyl thioxanthone (ITX) was used,^10^ which proved to be depletable via TAD when using 532 or 660 nm light.^11,13,14^ Several other thioxanthones have been screened for TAD; however, ITX proved to be the most effective.^24,25^ Further, 7-diethylamino-3-thenoylcoumarin (DETC)^8^ was found to be efficient for STED and TAD and soon became the most frequently used radical starter for two-wavelength photoinhibition lithography to date. Detailed studies showed that depletion at 532 nm can be either STED or TAD or even a combination of both, depending on the pulse width of the depleting laser.^11−14^ The shorter the pulse width, the more effective STED is, while in the case of continuous wave (CW) depletion lasers, TAD plays a decisive role too. A depletion laser of 640 nm wavelength, which is outside the fluorescence spectrum of DETC and hence cannot induce STED, has, however, also been shown to induce efficient depletion. This is because DETC shows a pronounced triplet–triplet absorption at 640 nm.^13,14^

DETC is by far the most widely used starter for STED or TAD subdiffraction lithography. It also yields better results in direct comparison with ITX.^7,8^ However, DETC has one significant disadvantage when it comes to nanostructuring for bio-applications. Micro- and nanostructures written with DETC show substantial residual fluorescence in the green spectral range (500–600 nm). Hence, the green and yellow detection channels of a fluorescence or confocal microscope cannot be used in multiple staining experiments.^26^ This is a severe drawback because it is state of the art in biological and medical research to stain various cell organelles or different proteins with distinct colors in order to monitor different cell organelles or to record protein–protein interactions. If the important green channel is blocked by the autofluorescence of the micro- or nanostructure to which the cells or proteins are attached, it cannot be used for multiwavelength imaging. ITX shows blue autofluorescence,^11,24^ which may interfere with the blue channel of fluorescence microscopes in bioimaging. Hence, a STED or TAD starter that is as efficient as DETC but fluorescently “silent” in the polymerized structures throughout the visible spectral range would be of great help in three-dimensional optical nanolithography for biomedical applications.

Recently, 4,4 bis(diethylamino)benzophenone, also called Michler’s ethyl ketone (MEK), has been reported to be depletable via TAD.^14^ Similarly to DETC, it shows pronounced transient absorption in the red spectral range, which is quite typical also for similar benzophenones.^17,27^ It was shown that an additional 642 nm CW laser beam forming an ordinary focal PSF overlapping the excitation PSF can prevent polymerization.^14^ A line was written with 405 nm one-photon excitation, and the line was interrupted in the area where the 642 nm laser was additionally switched on. However, no bottle- or donut-shaped depletion PSF was used in that work, and only one-photon excitation was applied,^14^ which is unsuitable for three-dimensional structuring. Within another recent study on MPP (but without STED or TAD), the starters ITX, DETC, and MEK were compared, and it was shown that structures written with MEK show superior mechanical stability compared to those written with ITX or DETC.^28^

In the following study, we will prove that MEK is a two-photon starter that is similarly effective as DETC in writing nanostructures using a 660 nm CW TAD beam. With both starters, we achieve linewidths ≤ 40 nm both in the lateral and axial direction showing that they are similarly useful for TAD lithography. However, unlike DETC, MEK shows only a low autofluorescence in the green channel of a fluorescence or a confocal microscope, so this channel can also be used for imaging cells in multiple-color staining experiments.

## 2 Materials and Methods

We used a home-built dual-beam two-photon lithography setup similar to the one described before.^20^ To initiate the multiphoton polymerization, laser pulses of 780 nm (82 MHz repetition rate, 110 fs, FFS-tSHG, Toptica, Germany) were applied. TAD was performed via a continuous wave (CW) 660 nm laser (Opus, Laser Quantum, Germany). The intensity of the excitation beam was controlled by an acousto-optical modulator (MT110-A1.5-IR, AA Opto Electronic, France), while the intensity of the TAD beam was directly adjusted by the laser. All powers quantified in this paper are powers entering the back aperture of the objective lens. Both beams were combined, and an α-Plan Apochromat, 100×, NA = 1.46 oil immersion objective lens (Zeiss, Oberkochen, Germany) was used for focusing. Measurements for determining the depletability of the photostarters by TAD were carried out using an ordinary 660 nm focus confocalized with a 780 nm excitation focus. Subdiffractional patterning was carried out with a so-called “bottle-beam”-shaped, three-dimensional TAD focus, which nominally bears zero intensity in the center, a ring of 660 nm light in the focal plane, and two pronounced foci above and below the focal plane; see right inset in Figure 1. Such a bottle-shaped point spread function was achieved by inserting an annular phase delay of π (home-built) into the 660 nm beam.^19^ The PSFs shown in Figure 1 were measured via backscattering from gold nanospheres (60 nm diameter) on a coverslip covered with immersion oil. In practice, the central zero contains less than 0.1% of the intensity within the foci above and below the focal plane. Stage scanning was carried out by a three axes piezo stage (P 562.3CD, Physik Instrumente PI, Germany) with a bidirectional positioning accuracy of 2/2/4 nm (*x*/*y*/*z*) and a 200 μm travel range in each direction. The writing speed was 100 μm/s.

**Figure 2:**
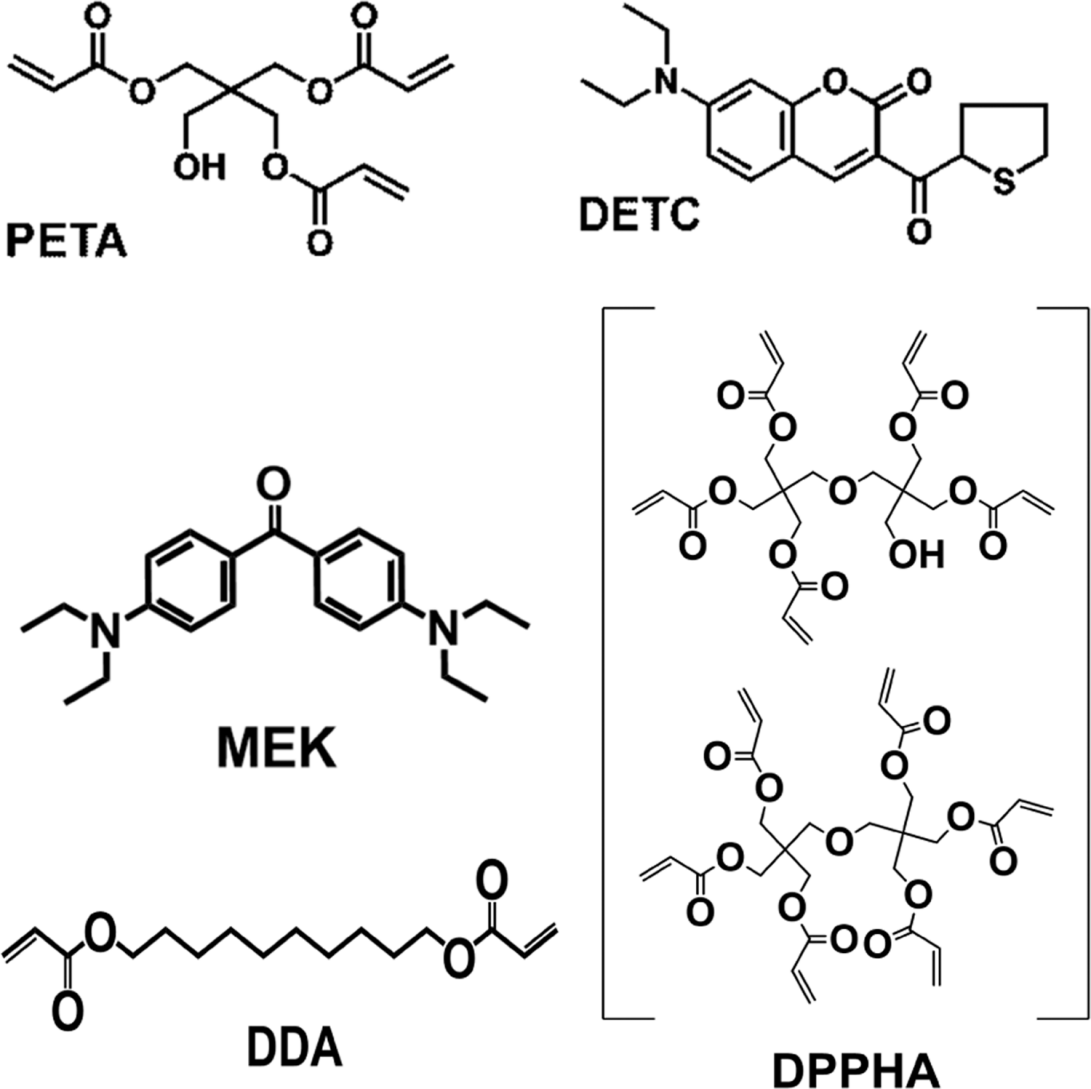
Structures of the used monomers (PETA or an 80/20 mixture of DPPHA and DDA) and the two starters DETC and MEK.

Two different types of acrylate monomers were used: either pentaerythritol triacrylate (PETA, Sigma-Aldrich)^10^ or an 80:20 (per weight) mixture of dipentaerythrit-penta-/hexa-acrylate (DPPHA, Sigma-Aldrich) and 1,10 bis(acryloyloxy) decane (DDA, TCI Chemicals), in short DPPHA:DDA.^29^ As starters, we used either DETC (Chemos, Germany; purity: 97%) or MEK (Sigma-Aldrich, purity: >97%). Figure 2 shows the chemical formulas of the monomers and the starters. All chemicals were used as received. Mutually combining the two acrylates with 0.25 wt % of one of the two starters results in four possible mixtures of resins: (PETA/DETC), (DPPHA:DDA/DETC), (PETA/MEK), and (DPPHA:DDA/MEK). The first combination (PETA/DETC) is a widely used resist for STED-inspired subdiffractional lithography.^7,8,20^

Scanning electron microscopy (SEM) images were obtained using a Zeiss 1540XB SEM after evaporating approx. 10 nm of gold. In order to determine the axial sizes of three-dimensional structures, SEM imaging was performed after tilting the samples by 60°. Photoluminescence (PL) spectra of the polymers were taken after forming thin films of the four composites and curing them under a UV lamp. For PL measurements, the films were excited by a 355 nm laser (1Q, CryLas, Germany), and the PL spectra were recorded with a fiber spectrometer (Quest X, B&W TEK). Atomic force microscopy (AFM) images were taken with a NanoWizard 3 (JPK Instruments, Berlin) using intermittent contact mode and PPP-NCHR cantilevers (Nanosensor, Switzerland). Gwyddion^30^ was used for image analysis.

HeLa cells were cultured in Dulbecco’s modified Eagle medium (DMEM, Thermo Fisher Scientific) supplemented with 10% fetal bovine serum (FBS, PAN-Biotech GmbH, Germany) and penicillin–streptomycin (100 IU/mL penicillin 100 μg/mL streptomycin) at 37 °C and 5% CO_2_. Before seeding, the 3D gratings made of PETA:DETC and PETA:MEK photoresists were washed in phosphate-buffered saline (PBS) for 24 h and subsequently coated with 50 μg/mL collagen in PBS (Ibidi, Germany) for 15 min. On average, 10,000 cells were seeded on the structures. 24 h after seeding, the cells were washed with prewarmed PBS and subsequently fixed using 4% paraformaldehyde in PBS for 20 min at room temperature, then permeabilized in 0.5% Triton X-100 with PBS for 10 min, blocked with 1% Albumin from chicken egg white (Sigma-Aldrich, Vienna, Austria), and stained for 20 min with 66 nM Alexa-488 conjugated to phalloidin.

Fluorescence and STORM images were acquired using a modified Olympus IX81 inverted epifluorescence microscope with an oil immersion objective lens (60×/1.42NA, Olympus, Vienna, Austria).^31^ For fluorescence microscopy, the samples were illuminated for 1 ms with 15.8 W/cm^2^ from a 488 nm excitation laser.

## 3 Results and Discussion

**Figure 3:**
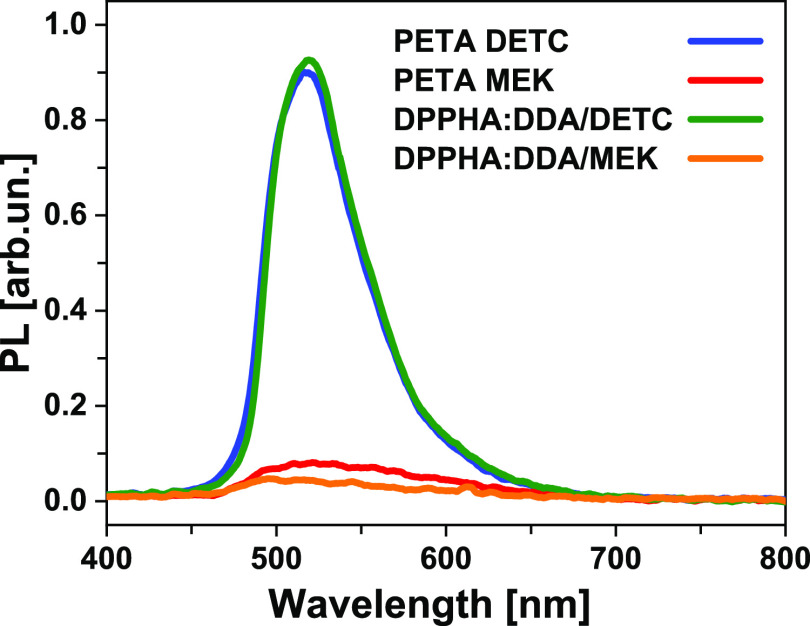
Photoluminescence of thin polymerized films of the four resins. The films were polymerized with a UV lamp. PL excitation was at 355 nm.

**Figure 4:**
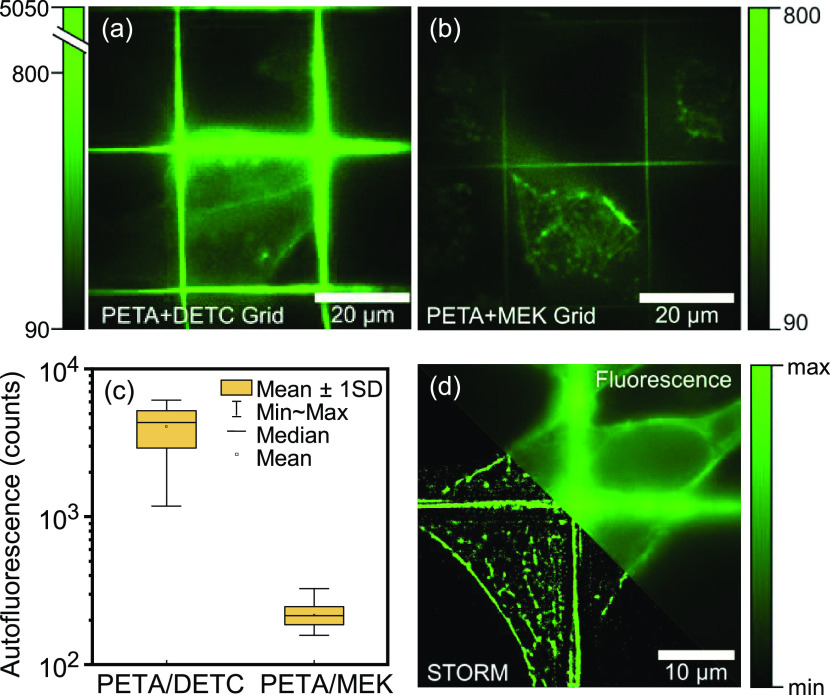
HeLa cells with actin stained with Alexa-488 on MPP gratings. (a) PETA/DETC grating and (b) PETA/MEK grating. The same staining protocol and the same excitation intensity (488 nm) were applied. The black/green lookup table in panel (a) is scaled such that the dynamics of the cell (90–800 counts) is linear, whereas counts beyond 800 up to 5050, stemming from the grating, are binned to bright green. No such binning was necessary for the cells on PETA/MEK shown in panel (b). (c) Statistics of the autofluorescence from the grids. 60 points on each grating were used for the statistics. (d) STORM image of cells on a PETA/MEK grating showing fine details of the actin cytoskeleton.

In an initial experiment, we took the PL spectra of all four mixtures. As we are interested in the autofluorescence of the final structures, it is important to take the spectra from the polymerized acrylates and not from a mixture of precursors or from the starters in solution. We polymerized thin films of each of the four resins using a UV lamp. The PL spectra are shown in Figure 3. Both resins containing DETC as a starter show the typical^11^ fluorescence of DETC between 480 and 630 nm with peaks at 517 nm (PETA/DETC) and 520 nm (DPPHA:DDA/DETC). In contrast, the polymers containing MEK show much less PL with peaks at 527 and 520 nm for PETA/MEK and DPPHA:DDA/MEK, respectively. Hence, MEK is the preferable starter whenever autofluorescence of the nanostructures must be minimized. This is further confirmed in Figure 4, where we imaged HeLa cells on top of two-photon polymerized gratings in a fluorescence microscope. The gratings were produced either from PETA/DETC (Figure 4a) or from PETA/MEK (Figure 4b). In both cases, the black-to-green lookup table was scaled to fit the fluorescence dynamics of the HeLa cells, which is 90 to 800 counts. In the case of PETA/DETC (Figure 4a), the grating shows a vastly overshooting autofluorescence of up to 5050 counts. These overshooting counts above 800 are all binned to bright green on the lookup table. Without such binning (i.e., scaling the lookup table linearly from 90 to 5050), the HeLa cells on PETA/DETC grids are barely visible. No such binning was necessary in the case of HeLa cells on PETA/MEK gratings (Figure 4b). Figure 4c shows the statistics of the autofluorescence from the two gratings. The fluorescence was evaluated at 60 different points on each grating to generate the statistics. For PETA/DETC and PETA/MEK, the medians are 4085 and 217 counts, respectively. The severe background and the stray light from the PETA/DETC grating make it impossible to perform super-resolved STORM imaging of cells on top of a PETA/DETC grating in the green channel of a fluorescence microscope. In contrast, STORM imaging of the HeLa cells on top of a PETA/MEK grating is readily facile, as shown in Figure 4d. (Stray-)photons from the grating do not overexpose the detector, and the fine details of the actin cytoskeleton are well-resolved.

**Figure 5:**
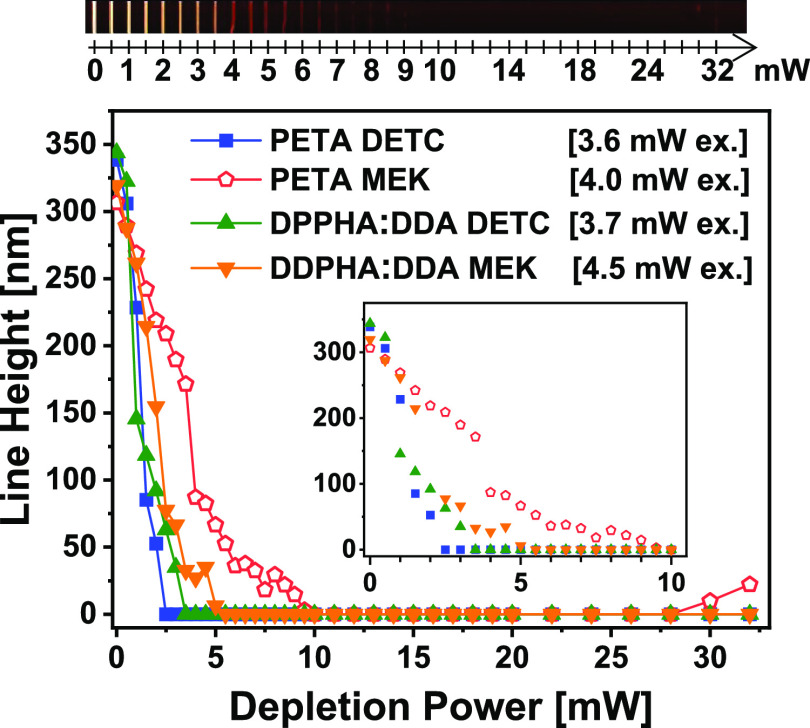
Line height as measured with AFM versus applied TAD power, using confocalized ordinary PSFs for excitation and depletion. On top, an exemplary AFM image is shown for (PETA/MEK). The depletion power was ramped up in steps of 0.5 mW up to 10 mW, followed by steps of 1 mW up to 20 mW, followed by 2  mW steps up to 32 mW. The excitation power, as given in the inserted legend for each resist, is chosen so that for 0 mW depletion power, the line height is between 300 and 350 nm. The inset shows the same data, zoomed out for the first 10 mW of depletion power.

Next, we characterized the depletion efficiency of the two starters. For this reason, we confocalized two ordinary PSFs for multiphoton excitation (780 nm) and for depletion (660 nm) and wrote a series of lines with increasing depletion power. The line height was characterized by AFM. One example is given in the upper part of Figure 5. More examples are given in the Supporting Information, Figures S1 and S2. For this example, we used (PETA/MEK) and adjusted the excitation power to 4.0 mW. The first vertical line at the left was written without 660 nm light (0 mW of depletion power). The line height, as measured with AFM, was 307 nm. The other lines were written while increasing the TAD power stepwise by 0.5 mW up to 10 mW, followed by steps of 1 mW up to 20 mW and 2 mW steps up to 32 mW. The height of the lines decreased with increasing TAD power, as depicted by the open red pentagons in Figure 5. From 9.5 mW up to 28 mW, no lines could be detected. Starting at 30 mW TAP power, faint lines appear, possibly induced by the joint action of the excitation and TAD lasers together. We repeated the experiment for the other three resists. In each case, we adjusted the MPP excitation intensity to achieve between 300 and 350 nm line heights for 0 mW depleting power. The respective excitation powers are 3.6 mW for (PETA/DETC), 3.7 mW for (DPPHA:DDA/DETC), and 4.5 mW for (DDPHA:DDA/MEK) as depicted in the legend in the graph of Figure 5. The TAD powers from where no polymerization took place are (see the zoomed inset of Figure 5) 2.5 and 3.5 mW in the case of (PETA/DETC) and (DPPHA:DDA/DETC), respectively, and 9.5 and 5.5 mW for (PETA/MEK) and (DPPHA:DDA/MEK), respectively. This means that the widely used resist (PETA/DETC) is most easily depleted with 660 nm among the four resists under investigation, and DETC as a starter is easier to be depleted than MEK; however, MEK is quite compatible. Further, we generally observed that for similar MPP excitation powers and 0 mW depletion, the resists started by MEK yield lower line heights than those started by DETC (see the Supporting Information). Hence, MEK is slightly inferior in terms of depletability, but it already starts with smaller lines when the same MPP excitation but no depletion is used. Further down, we will see that both effects seem to compensate for each other, and both starters are comparable in the achievable minimal width and height of suspended lines. Depletion experiments with different excitation powers than those used in Figure 5 are shown for all four resists in the Supporting Information, Figures S1 and S2.

**Figure 6:**
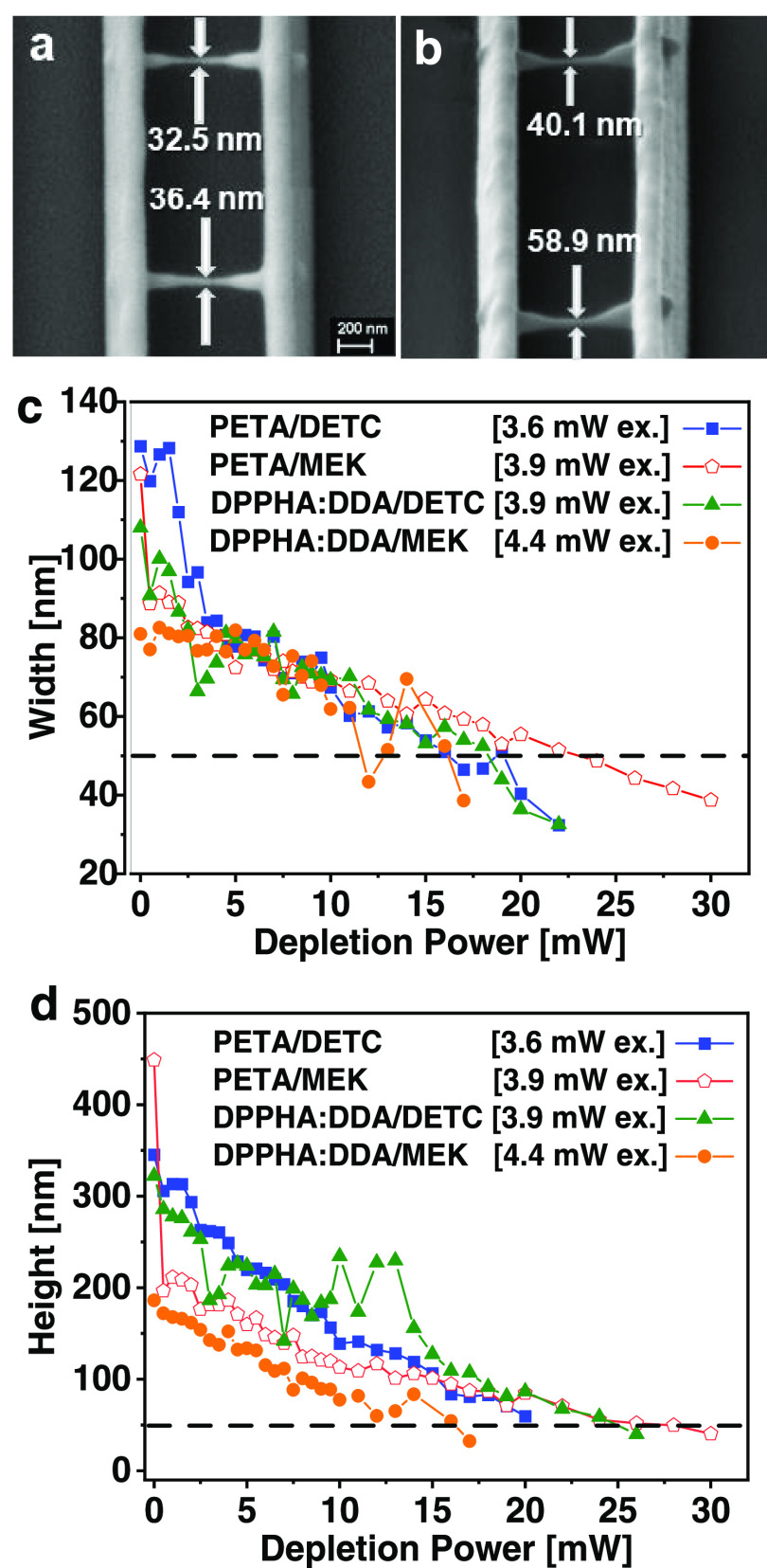
Two thick carrier lines (rails) were written in parallel without any depleting beam. Perpendicular to them, suspended lines were written with a TAD bottle-beam PSF of varying intensity. At the right and left ends of the suspended lines, the excitation intensity was increased by 50%, yielding thicker anchor points. (a) SEM top view of two such suspended lines (DPPHA:DDA/DETC). (b) SEM with sample tilted by 60° to evaluate the height of the suspended lines. (c) Measured linewidths as a function of TAD power at excitation powers given in the legend. (d) Axial height of the same lines, corrected by the perspective angle of the 60° sample tilt. The horizontal dashed lines in panels (c, d) represent the 50 nm “node,” which is clearly undercut in most cases. In panels (c, d), the TAD power was increased until no lines could be written anymore.

For the following experiments, we switched the annular π-phase mask into the TAD beam path in order to distribute the 660 nm TAD light around the focal spot, as shown by the PSF in the right inset of Figure 1. First, we blocked the TAD beam and wrote pairs of supporting rails, each about 250 nm wide and 1200 nm high and with a mutual distance of 1000 nm. These are the thick parallel lines in Figure 6a,b. Perpendicular to them, we wrote thin lines at the height of 900 nm so that they were suspended between the parallel rails and did not attach to the surface of the coverslip. Between writing the rails and writing lines, we waited about 5 min in order to reduce the impact of pre-excited/prepolymerized oligomers on the thickness of the lines. The spacing between the lines was 3 μm. While writing a thin line, the excitation power was initially increased by 50%, then set to the desired value, and then increased again. This renders two slightly thicker line ends attached to the two rails but thin lines in the center. The thick ends were necessary to assure firm attachment of the thin suspended lines to the rails. The center part of the thin lines was then evaluated using SEM after about 10 nm of gold evaporation. The top view in SEM (Figure 6a) allows us to determine the lateral structure size. In order to determine the vertical height of the suspended lines, the sample was tilted by 60° in SEM (Figure 6b).

Figure 6c,d shows the achieved widths and heights of the suspended lines, respectively, for all four resins. The excitation power used for each resin was adjusted such that without TAD, the narrowest yet reproducibly continuous lines could be achieved with MPP; these powers are given in the in-graph legend. We will start our discussion with the linewidths (Figure 6c). At no TAD intensity (pure MPP), we observe that the linewidths are all below the diffraction limit. This is due to the chemical nonlinearity of the polymerization threshold as a result of background oxygen in the resin, which is well-known for MPP lithography.^3,32^ At no or only weak TAD, we observe that the MPP linewidth is generally smaller in the case of MEK as a starter in comparison with DETC. For the PETA resist, the MPP linewidths written with MEK (red open pentagons, 123 nm without TAD) are slightly smaller than those written with DETC (blue squares, 129 nm without TAD), and for the DPPHA:DDA resist, the MPP linewidths written with MEK (orange circles, 81 nm without TAD) are substantially smaller than those written with DETC (green triangles, 108 nm without TAD). This finding is consistent with the observation mentioned above that the two-dimensional (2D) MPP lines written on the substrate using MEK are less in height than those using DETC when a similar MPP excitation power is used (also see the Supporting Information). When the TAD depletion power is increased up to the point where no continuous lines can be written anymore (these powers are different for each resist), the linewidths decrease for all four resists, meaning that both starters are effective for achieving substantially more narrow linewidths in TAD lithography compared to MPP lithography. The narrowest lines which could be achieved are 32 and 33 nm in cases of (PETA/DETC) and (DPPHA:DDA/DETC), respectively, and 39 nm for both resists using MEK as a starter. This means that both starters yield comparable linewidths, with a bit of an advantage for DETC because of its better depletability (Figure 5). Please note that a nominally 10 nm thick Au layer was evaporated prior to SEM inspection, and hence the true linewidths are probably even thinner than those measured with SEM. In the case of MEK, we also observed that using PETA as a resist monotonously lowers the linewidth (open red pentagons) and reliably yields sub-50 nm linewidths when TAD powers above 24 mW are used. However, using DPPHA:DDA as a resist (orange circles) yields a large scatter in experimental linewidth for depletion powers above 10 mW. We note that acrylates are prone to undergo some post-deposition shrinkage, which might contribute to the small size of the linewidths; however, it affects both MPP and TAD linewidths. The relative improvement from a (possibly shrunken) MPP linewidth to a (possibly shrunken) TAD linewidth is on the order of 66%. In order to evaluate whether shrinkage affects suspended lines more than lines that are written in direct contact with a support, we wrote lines directly on the glass substrate (covered by acryl-silanes) and retrieved linewidths of 38, 33, 45, and 38 nm for PETA/DETC, DPPHA:DDA/DETC, PEAT/MEK, and DPPHA:DDA/MEK, respectively, in very good agreement with the linewidths of the suspended lines (see the Supporting Information, Figure S4).

Figure 6d shows the results for the height of the suspended lines. The heights were measured from SEM images where the samples were tilted by 60° in the SEM, and the obtained values were corrected by a factor of 1/sin 60° = 1.15 in order to account for that perspective. However, we again have not corrected for 10 nm evaporated gold, so the line heights given in Figure 6d are conservative. Taking the line heights with no or only low TAD intensity, we observe that resins using MEK as a starter yield substantially lower line heights (around 200 nm, open red pentagons and orange dots) compared to resins containing DETC as a starter (300–350 nm, blue squares and green triangles). The data point for PETEA/MEK and 0 mW TAD is most possibly an outlier. Increasing the TAD power systematically reduces the line height in a quite monotonic fashion. Only in the case of DPPHA:DDA, but this time with DETC as a starter, the experimental line heights scatter a lot. This result, together with the finding that the linewidth of (DPPHA:DDA/MEK) was somewhat unpredictable above 10 mW of TAD power, leads us to conclude that PETA is the more reliable resist compared to DPPHA:DDA. A possible explanation is that there are higher variations in the local concentration of the constituents (monomers and starters) in the case of DPPHA:DDA compared to PETA-based resists, which is plausible because DPPHA:DDA itself is a mixture of two types of monomers, while PETA is homogeneous. Comparing the minimal achievable line heights using the two starters yields that MEK seems to be slightly advantageous: using PETA as a resist, we measured minimal line heights of 60 and 40 nm in the case of DETC or MEK as starters, respectively, and using DPPHA:DDA as a resist, we measured 40 and 32 nm minimal line heights, respectively.

## 4 Conclusions

We have compared four mixtures of photoresists that are usable in STED-inspired two-color photoinhibition lithography. In particular, we used a 660 nm CW laser for depletion, which is outside the photoluminescence spectrum of both starters, DETC and MEK, but induces transient absorption, which inhibits the photopolymerization of both acrylate monomers, PETA and DPPHA:DDA. To the best of our knowledge, this is the first report where MEK was used in subdiffractional TAD lithography. When using pure MPP lithography, MEK allows for writing more narrow structures compared to writing with DETC as a starter. However, DETC shows better depletability by TAD. In the end, both effects seem to compensate each other such that suspended lines of sub-40 nm diameter can be written with both of them. MEK shows much lower PL than DETC in both acrylates and is, therefore, for instance, better suited for writing nanoanchors for a single antibody attachment^26^ or protein fixation in flow cells.^33^ This is because the autofluorescence of DETC within the nanostructures disrupts the use of some of the color-channels of fluorescence, confocal, or super-resolving STORM microscopes. We further note that using 660 nm for depletion instead of the more common 532 nm also causes less chromatic aberration with respect to the 780 nm excitation beam, which is advantageous specifically for 3D patterning deeper inside the photoresist.^15^

## Supporting Information Available

Depletion matrices showing line heights as a function of excitation and depletion power, an SEM overview of lines suspended between pairs of rails and widths of supported lines (PDF)

## References

[ref1] Denk W.; Strickler J. H.; Webb W. W. Two-photon laser scanning fluorescence microscopy. Science 1990, 248, 73–76. 10.1126/science.2321027. 2321027

[ref2] Maruo S.; Nakamura O.; Kawata S. Three-dimensional microfabrication with two-photon-absorbed photopolymerization. Opt. Lett. 1997, 22, 132–134. 10.1364/OL.22.000132. 18183126

[ref3] Fischer J.; Wegener M. Three-dimensional optical laser lithography beyond the diffraction limit. Laser Photonics Rev. 2013, 7, 22–44. 10.1002/lpor.201100046.

[ref4] Klar T. A.; Wollhofen R.; Jacak J. Sub-Abbe resolution: from STED microscopy to STED lithography. Phys. Scr. 2014, T162, 014049 10.1088/0031-8949/2014/T162/014049.

[ref5] Hell S. W.; Wichmann J. Breaking the diffraction resolution limit by stimulated emission: stimulated-emission-depletion fluorescence microscopy. Opt. Lett. 1994, 19, 780–782. 10.1364/OL.19.000780. 19844443

[ref6] Klar T. A.; Hell S. W. Subdiffraction resolution in far-field fluorescence microscopy. Opt. Lett. 1999, 24, 954–956. 10.1364/OL.24.000954. 18073907

[ref7] Fischer J.; Ergin T.; Wegener M. Three-dimensional polarization-independent visible-frequency carpet invisibility cloak. Opt. Lett. 2011, 36, 2059–2061. 10.1364/OL.36.002059. 21633448

[ref8] Fischer J.; Wegener M. Three-dimensional direct laser writing inspired by stimulated-emission-depletion microscopy. Opt. Mater. Express 2011, 1, 614–624. 10.1364/OME.1.000614.

[ref9] Li L.; Gattass R. R.; Gershgoren E.; Hwang H.; Fourkas J. T. Achieving λ/20 Resolution by One-Color Initiation and Deactivation of Polymerization. Science 2009, 324, 910–913. 10.1126/science.1168996. 19359543

[ref10] Fischer J.; von Freymann G.; Wegener M. The Materials Challenge in Diffraction-Unlimited Direct-Laser-Writing Optical Lithography. Adv. Mater. 2010, 22, 3578–3582. 10.1002/adma.201000892. 20593434

[ref11] Wolf T. J. A.; Fischer J.; Wegener M.; Unterreiner A. N. Pump-probe spectroscopy on photoinitiators for stimulated-emission-depletion optical lithography. Opt. Lett. 2011, 36, 3188–3190. 10.1364/OL.36.003188. 21847203

[ref12] Fischer J.; Wegener M. Ultrafast Polymerization Inhibition by Stimulated Emission Depletion for Three-dimensional Nanolithography. Adv. Mater. 2012, 24, OP65–OP69. 10.1002/adma.201103758. 22323275

[ref13] Harke B.; Bianchini P.; Brandi F.; Diaspro A. Photopolymerization Inhibition Dynamics for Sub-Diffraction Direct Laser Writing Lithography. ChemPhysChem 2012, 13, 1429–1434. 10.1002/cphc.201200006. 22392895 PMC3491630

[ref14] Harke B.; Dallari W.; Grancini G.; Fazzi D.; Brandi F.; Petrozza A.; Diaspro A. Polymerization Inhibition by Triplet State Absorption for Nanoscale Lithography. Adv. Mater. 2013, 25, 904–909. 10.1002/adma.201204141. 23303534 PMC3594812

[ref15] He M.; Zhang Z.; Cao C.; Qiu Y.; Shen X.; Zhou G.; Cai Z.; Sun X.; He X.; Xu L.; Liu X.; Ding C.; Cao Y.; Kuang C.; Liu X. Single-color peripheral photoinhibition lithography of nanophotonic structures. PhotoniX 2022, 3, 25 10.1186/s43074-022-00072-2.

[ref16] Liaros N.; Gutierrez-Razo S. A.; Thum M. D.; Ogden H. M.; Zeppuhar A. N.; Wolf S.; Baldacchini T.; Kelley M. J.; Petersen J. S.; Falvey D. E.; Mullin A. S.; Fourkas J. T. Elucidating complex triplet-state dynamics in the model system isopropylthioxanthone. iScience 2022, 25, 103600 10.1016/j.isci.2021.103600. 35005547 PMC8717599

[ref17] Mondal J. A.; Ghosh H. N.; Ghanty T. K.; Mukherjee T.; Palit D. K. Twisting Dynamics in the Excited State of Michler’s Ketone. J. Phys. Chem. A 2006, 110, 3432–3446. 10.1021/jp0555450. 16526622

[ref18] Müller P.; Müller R.; Hammer L.; Barner-Kowollik C.; Wegener M.; Blasco E. STED-Inspired Laser Lithography Based on Photoswitchable Spirothiopyran Moieties. Chem. Mater. 2019, 31, 1966–1972. 10.1021/acs.chemmater.8b04696.

[ref19] Klar T. A.; Jakobs S.; Dyba M.; Egner A.; Hell S. W. Fluorescence microscopy with diffraction resolution barrier broken by stimulated emission. Proc. Natl. Acad. Sci. U.S.A. 2000, 97, 8206–8210. 10.1073/pnas.97.15.8206. 10899992 PMC26924

[ref20] Wollhofen R.; Katzmann J.; Hrelescu C.; Jacak J.; Klar T. A. 120 nm resolution and 55 nm structure size in STED-lithography. Opt. Express 2013, 21, 10831–10840. 10.1364/OE.21.010831. 23669940

[ref21] He X.; Li T.; Zhang J.; Wang Z. STED Direct Laser Writing of 45 nm Width Nanowire. Micromachines 2019, 10, 726 10.3390/mi10110726. 31661815 PMC6915467

[ref22] Cao C.; Qiu Y.; Guan L.; Wei Z.; Yang Z.; Zhan L.; Zhu D.; Ding C.; Shen X.; Xia X.; Kuang C.; Liu X. Dip-In Photoresist for Photoinhibited Two-Photon Lithography to Realize High-Precision Direct Laser Writing on Wafer. ACS Appl. Mater. Interfaces 2022, 14, 31332–31342. 10.1021/acsami.2c08063. 35786857

[ref23] Zhu D.; Xu L.; Ding C.; Yang Z.; Qiu Y.; Cao C.; He H.; Chen J.; Tang M.; Zhan L.; Zhang X.; Sun Q.; Ma C.; Wei Z.; Liu W.; Fu X.; Kuang C.; LI H.; Liu X. Direct laser writing breaking diffraction barrier based on two-focus parallel peripheral-photoinhibition lithography. Adv. Photonics 2022, 4, 066002 10.1117/1.AP.4.6.066002.

[ref24] Chi T.; Somers P.; Wilcox D. A.; Schuman A. J.; Iyer V.; Le R.; Gengler J.; Ferdinandus M.; Liebig C.; Pan L.; Xu X.; Boudouris B. W. Tailored Thioxanthone-Based Photoinitiators for Two-Photon-Controllable Polymerization and Nanolithographic Printing. J. Polym. Sci., Part B: Polym. Phys. 2019, 57, 1462–1475. 10.1002/polb.24891.

[ref25] Chi T.; Somers P.; Wilcox D. A.; Schuman A. J.; Johnson J. E.; Lisang Z.; Pan L.; Xu X.; Boudouris B. W. Substituted Thioxanthone-Based Photoinitiators for Efficient Two-Photon Direct Laser Writing Polymerization with Two-Color Resolution. ACS Appl. Polym. Mater. 2021, 3, 1426–1435. 10.1021/acsapm.0c01291.

[ref26] Wiesbauer M.; Wollhofen R.; Vasic B.; Schilcher K.; Jacak J.; Klar T. A. Nano-Anchors with Single Protein Capacity Produced with STED Lithography. Nano Lett. 2013, 13, 5672–5678. 10.1021/nl4033523. 24111646

[ref27] Singh A. K.; Ramakrishna G.; Ghosh H. N.; Palit D. K. Photophysics and Ultrafast Relaxation Dynamics of the Excited States of Dimethylaminobenzophenone. J. Phys. Chem. A 2004, 108, 2583–2597. 10.1021/jp037132+.

[ref28] Rajamanickam V. P.; Ferrara L.; Toma A.; Zaccaria R. P.; Das G.; Di Fabrizio E.; Liberale C. Suitable photo-resists for two-photon polymerization using femtosecond fiber lasers. Microelectron. Eng. 2014, 121, 135–138. 10.1016/j.mee.2014.04.040.

[ref29] Arnoux C.; Konishi T.; Van Elslande E.; Poutougnigni E.-A.; Mulatier J.-C.; Khrouz L.; Bucher C.; Dumont E.; Kamada K.; Andraud C.; Bladeck P.; Banyasz A.; Monnereau C. Polymerization Photoinitiators with Near-Resonance Enhanced Two-Photon Absorption Cross-Section: Toward High-Resolution Photoresist with Improved Sensitivity. Macromolecules 2020, 53, 9264–9278. 10.1021/acs.macromol.0c01518.

[ref30] Necas D.; Klapetek P. Gwyddion: an open-source software for SPM data analysis. Cent. Eur. J. Phys. 2012, 10, 181–188. 10.2478/s11534-011-0096-2.

[ref31] Mayr S.; Hauser F.; Peterbauer A.; Tauscher A.; Naderer C.; Axmann M.; Plochberger B.; Jacak J. Localization Microscopy of Actin Cytoskeleton in Human Platelets. Int. J. Mol. Sci. 2018, 19, 1150 10.3390/ijms19041150. 29641438 PMC5979344

[ref32] Kawata S.; Sun H. B.; Tanaka T.; Takada K. Finer features for functional microdevices. Nature 2001, 412, 697–698. 10.1038/35089130. 11507627

[ref33] Buchegger B.; Tanzer A.; Posch S.; Gabriel C.; Klar T. A.; Jacak J. STED lithography in microfluidics for 3D thrombocyte aggregation testing. J. Nanobiotechnol. 2021, 19, 23 10.1186/s12951-020-00762-8. PMC7814651 33461577

