# Supplementary material for: Low-Fluorescence Starter for Optical 3D Lithography of Sub-40 nm Structures
Source: ACS Appl Opt Mater. 2023 May 12;1(5):945–51. doi: 10.1021/acsaom.3c00031 (PMC10226181; doi:10.1021/acsaom.3c00031)
Supplement: Supplementary file 1 — ot3c00031_si_001.pdf [file ot3c00031_si_001.pdf]

Low-fluorescent starter for optical 3D lithography of sub-40nm structures

Supporting Information

Georgii Gvindzhiliia,[1] Dmitry Sivun,[2] Christoph Naderer,[2] Jaroslaw Jacak,[2]

and Thomas A. Klar[1,*]

[1] Institute of Applied Physics, Johannes Kepler University Linz, 4040 Linz, Austria

[2] Department of Medical Engineering, University of Applied Sciences Upper Austria, 4020 Linz, Austria

* email: thomas.klar@jku.at

A Depletion Matrices

A.1 PETA monomer

Depletion matrices were taken with confocalized, ordinarily shaped excitation and TAD PSFs, in order to determine the depletion efficiency. Figure S1 shows AFM images of lines written with a 780 nm MPP excitation laser of powers as indicated. The 660 nm TAD powers started at 0 mW and were increased stepwise by 0.5 mW up to 10 mW, followed by 1 mW steps to 20 mW and by 2 mW steps up to 32 mW. The resist was PETA. In the left and right columns, DETC and MEK were used as starters, respectively. Each line was tethered tight to the glass slide at both ends using 1.5 times the nominal excitation power. These tethering points are still visible at high TAD powers, where no lines are visible anymore.

The line height at 0 mW depletion power shows the line height of ordinary MPP lithography. Sub-diffractional line heights are possible due to the chemical nonlinearity of the polymerization threshold. At the same excitation power, the line height is smaller when MEK is used as a starter compared to DETC being used as a starter. For instance, using 3.7 mW of excitation power, we measure a line height of 390 nm in case of DETC (first point of the green data set, lower left graph in Figure S1). The line height is only 81 nm in case of MEK for the same MPP excitation power (first point of the black data set, lower right graph). In case of 3.8 mW excitation powers, the pure MPP line heights are 425 and 213 nm for DETC and MEK as starters, respectively.

When the depletion power was switched on, the line heights decreased rapidly, and in case of DETC as the starter, no lines were measured in the AFM images for depletion powers from 1.0, 1.5, 2.5, 4.0, and 5.0 mW onwards for excitation powers of 3.4, 3.5, 3.6, 3.7, and 3.8 mW, respectively. Hence, we zoomed out the depletion power range from 0 to 10 mW in the lower left graph of Figure S1, because from 11 to 32 mW, all data points are 0 nm.

In case of MEK as the starter (lower right graph in Figure S1), a similar picture arises. The depletion powers from whereon no lines are detectable are 1.0, 2.0, 4.0, and 9.5 mW for excitation powers of 3.7, 3.8, 3.9, and 4.0 mW. However, for an excitation power of 4.1 mW, complete depletion of the polymerization could not be reached anymore. In case of 4.0 mW excitation power, lines were detectable again from 30 mW depletion power onward, probably due to the joint action of both beams.

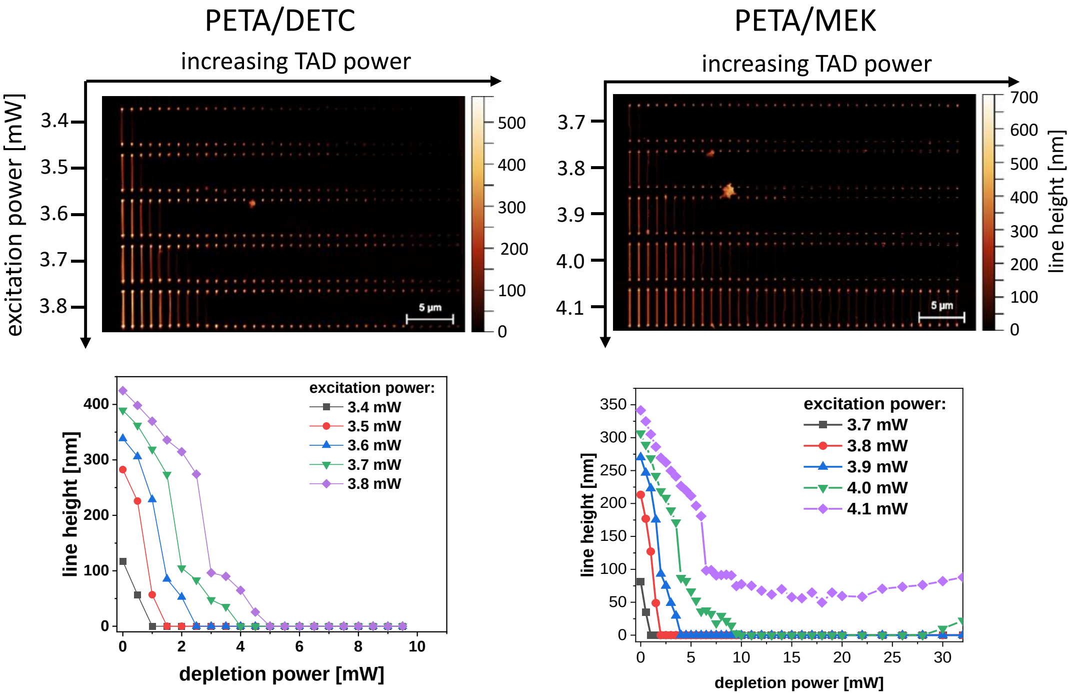

Figure S1. AFM images of PETA lines written with 780 nm MPP excitation powers as indicated and increasing TAD powers. The TAD powers started at 0 mW, where increased stepwise by 0.5 mW up to 10 mW, followed by 1 mW steps to 20 mW and by 2 mW steps up to 32 mW. In the left and right images, DETC and MEK were used as starters, respectively. The graphs below the AFM images show the measured line heights as a function of the TAD depletion power and the excitation power as indicated in the inserted legend.

A.2 DPPHA:DDA monomer mixture

Similar measurements were also carried out for DPPHA:DDA as the monomers and again with DETC and MEK as starters. The results are shown in Figure S2.

Sub-diffractional line heights are obtained due to the chemical nonlinearity of the polymerization threshold.

When the depletion power was switched on, the line heights again decreased rapidly, and in case of DETC as the starter, no lines were measured in the AFM images for depletion powers from 1.5, 2.5, 4.5, 5.0, and 7.5 mW onwards for increasing powers as indicated, respectively. Again, we zoomed out the depletion power range from 0 to 10 mW in the lower left graph, because from 11 to 32 mW, all data points are 0 nm. In case of MEK as the starter (lower right), no lines were measured in the AFM images for depletion powers from 1.0, 2.5, 3.0, 5.5, and 9.0 mW onwards for increasing excitation powers as indicated. This time, even for the highest excitation power of 4.6 mW, a complete depletion was achieved and no lines were measured above 10 mW.

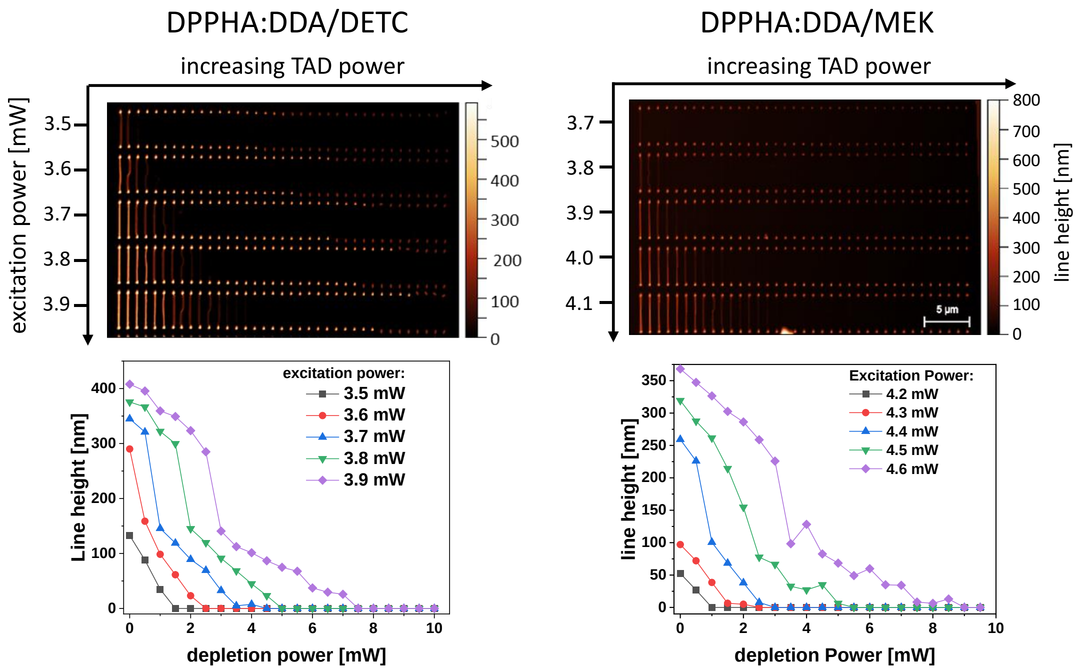

Figure S2. AFM images of DPPHA:DDA lines written with 780 nm MPP excitation powers as indicated and increasing TAD powers. The TAD powers started at 0 mW, where increased stepwise by 0.5 mW up to 10 mW, followed by 1 mW steps to 20 mW and by 2 mW steps up to 32 mW. In the left and right images, DETC and MEK were used as starters, respectively. The graphs below the AFM images show the measured line heights as a function of the TAD depletion power and the excitation power as indicated in the inserted legend.

B SEM overview of lines suspended between pairs of rails

Figure S3 shows a total-view-SEM of TAD-lines written with the resist (PETA/DETC) as an example. Four pairs of lines ("rails") were written with MPP as described in the main text. Perpendicular to them, thin lines were written with TAD lithography. Within each pair of rails, the same MPP excitation power was used as indicated to the left of the SEM image. From left to right, the TAD power was increased along the rails, as indicated below the SEM image. Above some maximal TAD power, depending on the excitation power, no lines could be written. For the detailed evaluation, zoomed SEM images were taken as those two shown in Figure 6a,b of the main manuscript.

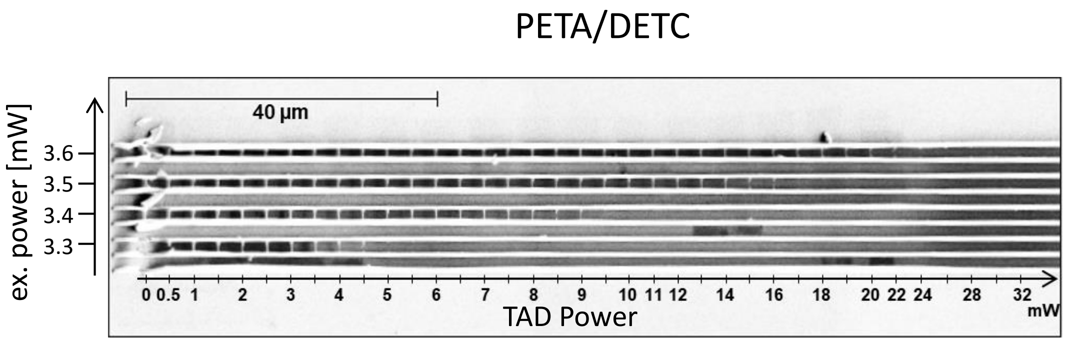

Figure S3. Large scale SEM overview of the TAD lithography experiment using different MPP excitation and TAD depletion powers. A bottle-beam TAD PSF was applied.

C Width of supported lines

In order to evaluate, whether shrinkage affects suspended lines more than lines which are written in direct contact with a support, we wrote lines directly on the glass substrate (covered by acryl-silanes) and retrieved line widths of 38, 33, 45 and 38 nm for PETA/DETC, DPPHA:DDA/DETC, PEAT/MEK and DPPHA:DDA/MEK, respectively (Figure S4), in very good agreement with the linewidths of the suspended lines (see Figure 6c). The excitation and TAD powers used for writing these supported lines are:

for PETA/DETC - 3.1mW of excitation and 16mW of TAD,

for PETA/MEK - 3.2 mW of excitation and 17mW of TAD,

for DPPHA:DDA/DETC - 2.7mW of excitation and 14mW of TAD,

for DPPHA:DDA/MEK - 3.6mW of excitation and 12 mW of TAD.

For these measurements, an acoustic-optical modulator from ISOMET, USA was used to control the excitation intensity.

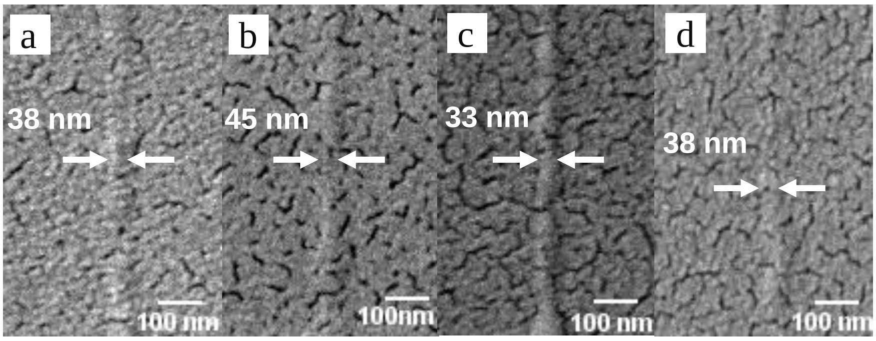

Figure S4. SEM images of a) PETA/DETC; b) PETA/MEK; c)DPPHA:DDA/DETC; d) DPPHA:DDA/MEK lines written directly on the glass substrate. The lines where covered by 10 nm of gold, so the given widths are conservative estimates.
